# Bilateral Sertoli Cell Tumors in a Patient with Androgen Insensitivity Syndrome

**DOI:** 10.1155/2017/8357235

**Published:** 2017-03-13

**Authors:** Roberta Fonseca de Souza, Janaina Pereira da Silva, Bruno Vieira Balla, Rodrigo Neves Ferreira, Antônio Chambô Filho

**Affiliations:** ^1^Department of Obstetrics and Gynecology, Santa Casa de Misericórdia, Vitória, ES, Brazil; ^2^Santa Casa de Misericórdia, Vitória, ES, Brazil

## Abstract

Androgen insensitivity syndrome is the most common cause of male pseudohermaphroditism and the third most common cause of primary amenorrhea. This genetic alteration is a consequence of inherited defects on the X chromosome causing total or partial damage to the intrauterine virilization process due to functional abnormalities in the androgen receptors. The present report describes a 22-year-old patient with a female phenotype and a 46, XY karyotype, presenting with bilateral inguinal tumors. The tumors were surgically removed at the* Santa Casa de Misericórdia* Hospital in Vitória, Espírito Santo, Brazil. Pathology revealed bilateral testicles with Sertoli cell tumors. According to the international literature, prophylactic gonadectomy following puberty is recommended due to the progressive risk of neoplastic transformation in the residual gonads.

## 1. Introduction

Complete androgen insensitivity syndrome, formerly known as testicular feminization, was first described in detail in 1953 in a classic report of 82 cases published by Morris [1]. Since this disease is rare, there are no statistics on its actual prevalence in the population; however, it is estimated to occur in around 1 in 20,400 to 1 in 99,100 male infants [2].

The androgens synthesized in the gonads during embryonic life are of vital importance in determining the male phenotype in an embryo with the 46, XY karyotype. Structural changes in the hormone receptor as a consequence of possible mutations in the X chromosome prevent it from functioning, leading to the expression of a female phenotype in an individual with a male karyotype. All the sex characteristics develop completely; however, the internal genitalia are absent, since production of the anti-Müllerian hormone is maintained. Anti-Müllerian hormone is produced by the Sertoli cells; therefore, detection of this hormone in serum suggests the presence of testicles. In the case of complete androgen insensitivity syndrome (CAIS), serum levels are high, while, in other intersex states, levels may be below normal or undetectable. Therefore, measuring anti-Müllerian hormone levels is of key importance in the diagnosis of male pseudohermaphroditism [3, 4].

The clinical signs that characterize the disease consist of female behavior, complete lack or scarcity of axillary and pubic hair, adequate female breast development, female external genitalia, blind vaginal pouch, absence of internal genitalia, and the presence of gonads with seminiferous tubules; however, spermatogenesis is absent [4, 5].

Individuals with androgen insensitivity syndrome should be submitted to prophylactic gonadectomy due to the risk of developing testicular malignancy. Studies in adults have shown that 50% of testicles containing a carcinoma in situ develop an invasive tumor within five years of diagnosis when left untreated. The question under debate is the ideal moment for surgery [6, 7].

Historically, surgery was performed as soon as possible to avoid uncomfortable psychosexual questions during adolescence or at the beginning of adult life. More recently, surgery at the end of the teenage years or in the early twenties has been preferred; nevertheless, when opting for this management, it should be remembered that the prevalence of carcinoma in situ is between 2 and 5% during puberty [7, 8].

The present report describes the histopathology findings of bilateral Sertoli cell tumors in a patient with androgen insensitivity syndrome receiving care at the Department of Gynecology and Obstetrics,* Santa Casa de Misericórdia*, Vitória, Espírito Santo, Brazil.

The internal review board of the* Santa Casa de Misericórdia* Hospital approved the procedures involved in this report on April 27, 2016, under reference number CAAE 51407815.2.0000.5065. Written informed consent for the publication of this report and corresponding images was obtained from the patient.

## 2. Case Report

An unmarried, white 22-year-old patient with a female phenotype, living in a small town in the state of Espírito Santo, presented at the gynecology outpatient clinic of the* Santa Casa de Misericórdia* Hospital on April 25, 2014 reporting primary amenorrhea. She had begun her sexual life at 19 years of age and had never conceived. She reported having been monitored by a multidisciplinary medical team since she was 17 years old.

The patient had a history of hypothyroidism and was undergoing treatment with 100 mcg/day of levothyroxine. There was a positive family history of malformation of the genital tract (two aunts with uterine agenesis). The patient was 1.70 meters in height and weighed 52 kg. Physical examination showed her to be in good general health, with good coloring, well-hydrated, acyanotic, and anicteric and with no swelling. She had poorly developed secondary sex characteristics, small breasts and sparse body hair (vulva and axillae). Her cardiovascular and respiratory systems were normal. Abdominal palpation revealed two inguinal masses, both approximately 4.0 cm in size, mobile and painless.

Gynecological examination showed a trophic vulva, sparse pubic hair, and no lesions or dyschromia. Pelvic examination revealed a vaginal canal about 7 cm in length with normal elasticity and rugae, no apparent lesions, and blind vaginal pouch. Bimanual palpation revealed absence of the cervix.

The patient had brought the results of imaging exams. Magnetic resonance imaging (MRI) showed normal bone morphology with normal signal intensity patterns; no uterus and no ovaries; bladder of normal size with regular walls; and partial vaginal canal with the upper segment missing. There was no free fluid in the abdominal cavity. Nodular formations with regular contours and well-defined borders were located at the level of the inguinal canal, bilaterally and symmetrically, measuring around 3.4 × 2.4 × 1.4 on the right side and 3.6 × 2.5 × 1.4 on the left side, with a slight bulging of the corresponding subcutaneous tissue. Clinically, this finding could correspond to ectopic gonads (cryptorchidism). Ultrasound of the urinary tract was normal. Pelvic ultrasonography showed absence of a uterus at the usual site (agenesis). The ovaries were not visualized, and there was no sign of tumors in the ovarian region or in the pelvis. No free fluid was detected in the pelvis. Previous laboratory tests showed FSH 12.0 mIU/mL, LH 52.5 mIU/mL, TSH 1.092 *μ*IU/mL, cortisol 24.27 *μ*g/dL, 17-hydroxyprogesterone 198 ng/dL, estradiol 33 pg/mL, and 8:00 a.m. cortisol 23.99 *μ*g/dL.

G-banded karyotype was performed on a peripheral blood sample, with results showing the 46, XY genotype.

**Figure 1:**
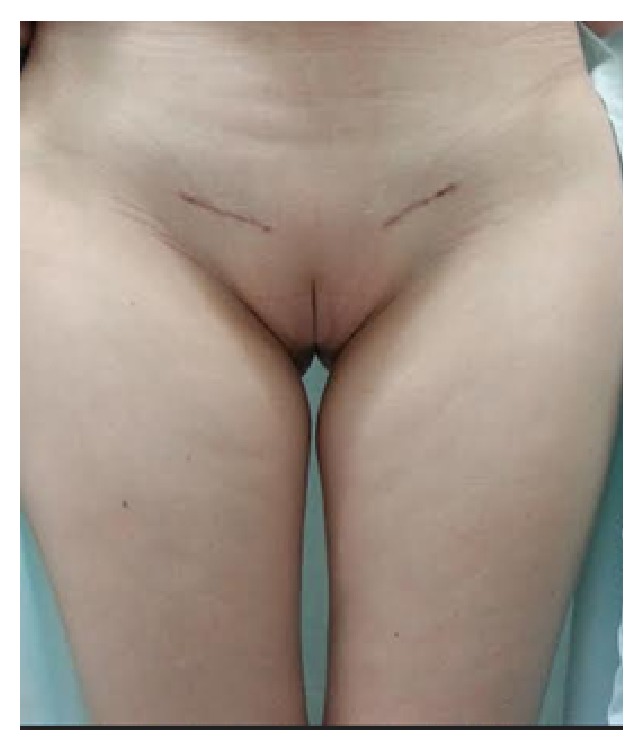
Surgical outcome following bilateral incisions for gonadectomy.

**Figure 2:**
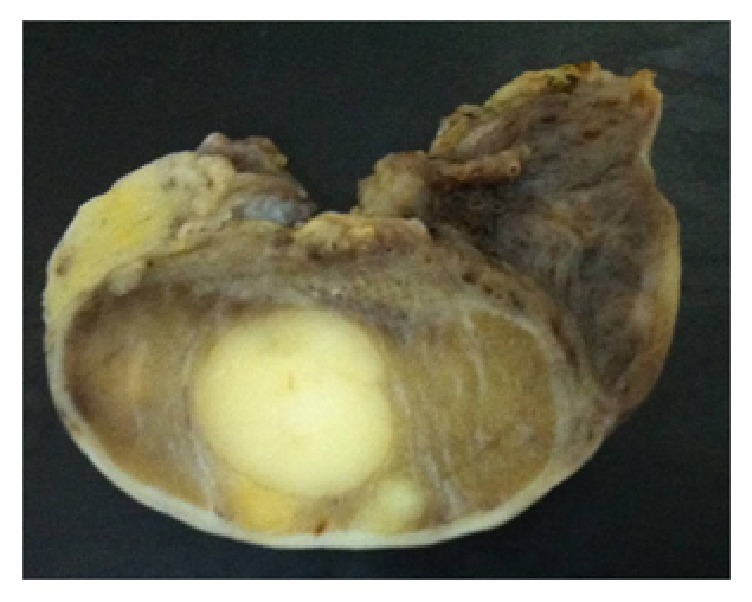
Macroscopy: a solid nodule in the testicular parenchyma.

**Figure 3:**
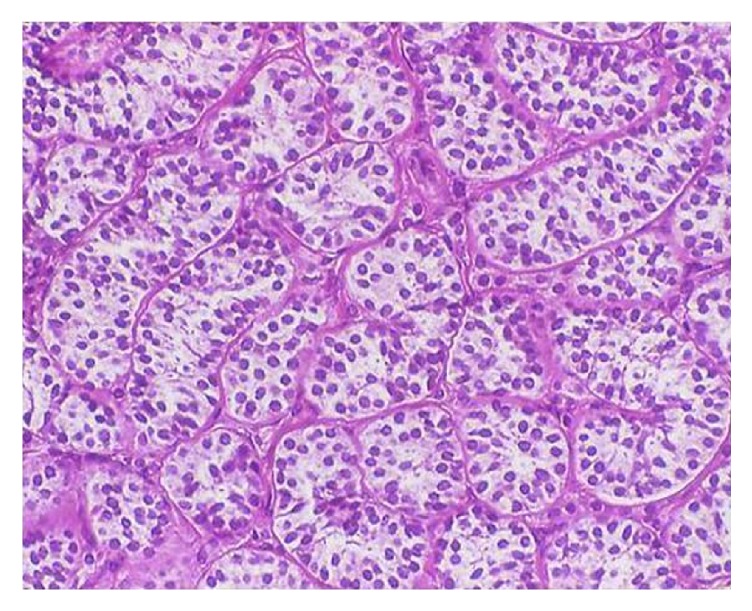
Microscopy: Sertoli cell tumor, with Sertoli cell tubules under high magnification (HE staining, magnification 400x).

The patient was referred to the hospital's Department of Gynecology for bilateral gonadectomy (Figure 1). The procedure was uneventful and the surgical specimens were sent for anatomopathology, with results showing 3 Sertoli cell tumors in the right gonad, the largest tumor measuring 1 cm in diameter and a Sertoli cell tumor measuring 1 cm in diameter in the left gonad (Figure 2). In all cases, the tumors had the same histological pattern and there were no signs of malignancy (Figure 3).

The patient is being monitored clinically at the gynecological endocrinology clinic and is currently under hormone replacement therapy with 2 mg of estradiol valerate (Primogyna®, Schering, Brazil).

## 3. Discussion

Androgen insensitivity syndrome is a metabolic disease characterized by complete resistance to the effects of androgens. According to the literature, its prevalence ranges from 1 in 20,400 to 1 in 99,100 male infants and the disorder is associated with X-linked recessive inheritance [1, 3].

Although diagnosis is usually reached after puberty, it is known that 1-2% of the children who present with an inguinal hernia or a palpable lump in this region may have alterations associated with the syndrome [3]. In adolescence, the principal complaint is primary amenorrhea, with androgen insensitivity syndrome being the third most common cause of this alteration [5]. Diagnosis is based on findings of a female phenotype, absence of the upper genital tract, and confirmation of the 46, XY karyotype. The principal complaint of the patient in question was primary amenorrhea, and, at physical examination, two palpable masses were found in both inguinal regions, in addition to clinical signs of androgen resistance.

Pelvic examination and evaluation of the gonads can be made by supplementary tests such as ultrasonography and MRI, which, according to the review published by Khan and Craig, are the most accurate tests in such cases [6]. Ultrasound is ideal for an initial diagnostic approach, since it is cheap and easily accessible and its sensitivity for detection of the gonads is good. However, MRI is considered the best option, since it provides a more detailed evaluation and can be used to complement ultrasound findings [6].

In the case presented here, both ultrasonography and MRI revealed the fact that the upper genital tract was absent and that the gonads were missing at the usual site. Compared to ultrasonography, MRI provided a more precise definition of the site and size of the ectopic testicles. Neither of the two methods identified any malformation of the urinary tract.

The incidence of testicular cancer is higher in developed countries, with the disease affecting 1.5/100,000 inhabitants worldwide. Female patients diagnosed with Morris syndrome may develop gonadal tumors. According to the literature, the risk of these patients developing malignant tumors is distinctly higher than that of the general population [9–11]. Manuel et al. reported that the risk of malignancy is accumulative and increases with age, with the risk in patients of 25 and 50 years of age being 3.6% and 33%, respectively [10]. Nonetheless, the risk of malignancy in childhood remains low, so bilateral prophylactic gonadectomy is recommended only after puberty, since by then the patient will have benefited from the spontaneous development of the secondary sex characteristics and better bone maturation due to gonadal hormone production [12, 13].

The malignant tumors often associated with this syndrome are seminomas and gonadoblastomas, although other histological forms such as teratomas, choriocarcinomas, yolk sac tumors, and embryonal tumors may also be present. Likewise, benign tumors such as adenomas, Leydig cell, and/or Sertoli cell tumors are other possible findings [14–16].

In the case described here, prophylactic gonadectomy was performed after puberty and the histopathology finding of Sertoli cell tumors in both gonads was unusual. The prevalence of this type of benign tumor is low in the population, representing 0.4 to 1.5% of testicular tumors in adults and 4% in children [16, 17].

Treatment is not restricted to just removing the gonads but demands multidisciplinary management in an attempt to minimize the psychological impact, since there is discordance between the individual's 46, XY genotype and female phenotype [18, 19]. Guidance with respect to sexuality and the impossibility of reproduction is also important aspects and cannot be neglected. In this case, the patient received appropriate counseling and had no complaints regarding her sexual life. Bearing this in mind, expectant management was adopted.

Following surgery, estrogen replacement therapy is recommended [20]. Some studies show that estrogen is beneficial in maintaining bone mass, preserving secondary sex characteristics, and preventing the symptoms of hypoestrogenism. Adding progesterone to estrogen therapy during hormone replacement therapy is unnecessary, since the patient has no uterus. Although there are doubts regarding the most appropriate dose and route of administration, either natural or synthetic estrogens can be used, either orally or transdermally [20, 21]. Therefore, taking into consideration the questions of compliance and access to the medication, it was decided to use oral estrogens for hormone replacement therapy. Following treatment initiation, the patient remains asymptomatic and is being followed up at the gynecological endocrinology outpatient clinic of the* Santa Casa de Misericórdia* Hospital in Vitória.
